# Verification of Reinforced Surface Loose Layer of Zinc–Aluminum–Magnesium Steel Plate

**DOI:** 10.3390/ma16186221

**Published:** 2023-09-15

**Authors:** Junxue Chen, Zheng Chen, Junjiao Yang

**Affiliations:** 1 College of Chemistry, Beijing University of Chemical Technology, Beijing 100029, China; cnrublab@cnrublab.com; 2 Analysis and Test Center, Beijing University of Chemical Technology, Beijing 100029, China; chenzheng@buct.edu.cn

**Keywords:** zinc–aluminum–magnesium steel plate, loose layer, silane coupling agent

## Abstract

The corrosion resistance of zinc–aluminum–magnesium steel plates (Zn–Al–Mg steel plates) is significantly higher than that of galvanized steel plates. However, the unsatisfactory bonding performance of Zn–Al–Mg steel plates significantly limits their widespread application. In this study, X-ray photoelectron spectroscopy is employed to detect changes in the surface oxygen content of Zn–Al–Mg steel plates after different temperature treatments to confirm the existence of surface loose layers. In particular, changes in the surface oxygen content of the Zn–Al–Mg steel plates after the oxide layer is removed are investigated under saturated H_2_O vapor and O_2_ environmental conditions, and the cause of the formation of loose surface layers is determined. The uneven distribution of elements on the surface of the Zn–Al–Mg steel plates is investigated with scanning electron microscopy and energy dispersive spectroscopy. Nuclear magnetic resonance is employed to determine the size of the network spatial structure formed by silane coupling agents under different hydrolysis conditions and to further investigate the bonding performance of hydrolysate-modified Zn–Al–Mg steel plates. Several typical automotive adhesives are utilized to compare and examine the changes in the tensile strength of the Zn–Al–Mg steel plate bonding before and after modification with the silane coupling agent and analyze the structural damage of the adhesive at the bonding interface. The results confirm that the silane coupling agent strengthens the loose layer on the surface of the Zn–Al–Mg steel plate.

## 1. Introduction

Zinc–aluminum–magnesium steel plates (Zn–Al–Mg steel plates) are a novel type of steel plate composed of materials with low density, high specific strength and stiffness, strong corrosion resistance, high edge protection performance, and high impact resistance [1,2,3,4,5,6]. These plates have a stronger corrosion resistance than ordinary galvanized sheets, and their coating can be thinner, which contributes to their light weight. Therefore, Zn–Al–Mg steel plates have been extensively investigated [7,8,9,10,11,12,13,14,15]. Zn–Al–Mg steel plates are widely utilized in the photovoltaic industry, equipment manufacturing, home appliance manufacturing, construction, aerospace, high-speed rail, and automotive manufacturing and are vital to the development of modern industries. However, compared with galvanized steel sheets, Zn–Al–Mg steel sheets exhibit unsatisfactory adhesion, limiting their application in the automotive field.

Most studies on Zn–Al–Mg steel plates focus on their anti-corrosion performance, whereas few focus on increasing their adhesion performance through surface modification methods [16,17,18,19,20]. The light weight of automobiles does not affect their safety performance; therefore, several bonding materials are utilized in their manufacturing process. However, the mechanism of bonding interactions between automotive adhesives and steel plates remains unclear. Studies on improving the bonding performance of Zn–Al–Mg steel plates via surface modification are few, whereas the bonding performance of steel plates must be urgently improved via mechanical methods.

Zn, Al, and Mg are highly active metals that undergo oxidation reactions upon contact with air to generate hydroxides and oxides. A microstructure composed of hydroxides and oxides is formed on the surface of Zn–Al–Mg steel plates, thus resulting in numerous hydroxyl groups on the surface of Zn–Al–Mg steel plates [21]. Most automotive adhesives are of the epoxy type, and active groups such as hydroxyl, carboxyl, and amino groups undergo ring-opening reactions with the epoxy groups [22,23,24,25,26]. During the curing process, these adhesives may undergo an epoxy ring-opening curing reaction with hydroxyl groups on the steel plate surface. In addition, the curing agent of the epoxy adhesive is composed primarily of amines, and the amino group can interact with the hydroxyl groups on the surface of the steel plate through the van der Waals interaction, thereby forming a bonding anchor on the surface of the steel plate to enhance the interfacial force. Hence, Zn–Al–Mg steel plates exhibit high bonding strength and are suitable as automotive steel plates. However, interface detachment problems typically occur during their actual usage, implying a loose layer of hydroxide on their surface.

Silane coupling agents have unique chemical structures. They contain organic and inorganic functional groups that can form a “molecular bridge” between the interface of inorganic compounds and organic matter, thus connecting two materials of different structural properties and with significant affinity differences [27,28,29,30]. Silane coupling agents are typically adopted as surface treatment agents, crosslinking agents, tackifiers, and sealants. They are widely utilized to treat metal surfaces and are excellent surface modification agents for Zn–Al–Mg steel plates [31,32,33,34,35]. Numerous silicon hydroxyl groups are generated during the hydrolysis of silane coupling agents, which can be grafted onto the surfaces of materials containing hydroxyl groups as active groups [36,37,38]. By controlling the silane coupling agent’s hydrolysis rate, the hydrolyzed molecule’s structure can form smaller molecular structural units, diffusing and penetrating the loose layer of Zn–Al–Mg steel plates. Under heating conditions, the silicon hydroxyl group undergoes a dehydration reaction with the hydroxide, thereby strengthening the structure of the loose layer [39]. The existence of a loose layer on the surface of the Zn–Al–Mg steel plate was studied and confirmed through X-ray photoelectron spectroscopy (XPS), scanning electron microscopy (SEM), scanning electron microscopy/energy dispersive spectroscopy (SEM-EDS), and other methods. The formation mechanism of the loose layer was verified through experiments in oxygen and water vapor atmospheres. The hydrolysis mechanism and molecular weight control of silane coupling agents were studied through nuclear magnetic resonance (NMR). After a silane coupling agent is grafted onto the surface of the steel plate, the functional groups on the silane coupling agent can undergo chemical reactions with the adhesive, thereby improving the interfacial bonding strength between the Zn–Al–Mg steel plate and adhesive. The surface modification of the Zn–Al–Mg steel plate improves its corrosion resistance, enhances its bonding performance, and expands its application range.

## 2. Experiment

### 2.1. Material and Instrument

The Zn–Al–Mg steel plate is a steel plate coated with a galvanized zinc–aluminum–magnesium alloy coating via hot dipping, with zinc, aluminum, and magnesium contents of 97, 1.5, and 1.5 wt%, respectively, and a coating thickness of approximately 5 μm. The chemicals utilized here are as follows: E51 epoxy resin (analytically pure, Beijing Chemical Plant, Beijing, China); hexahydrophthalic anhydride (analytically pure, Beijing Chemical Plant); 2,4,6-tris (dimethylaminomethyl) phenol (DMP30) (analytically pure, Beijing Chemical Plant); isoamyl acetate (analytically pure, Aladdin Company, Bay City, MI, USA); petroleum ether (analytical pure, Aladdin Company); acetone (analytically pure, Aladdin Company); ethanol (analytically pure, Aladdin Company); N-(β-Aminoethyl)-γ-Aminopropyl trimethoxysilane (KH792) (analytically pure, Aladdin Company); acetic acid (analytical pure, Beijing Chemical Plant); nitric acid (analytically pure, Beijing Chemical Plant); sodium hydroxide (analytically pure, Beijing Chemical Plant); and ammonia water (analytically pure, Fuchen Tianjin Chemical Co., Ltd., Tianjin, China). Ultrapure water was prepared with a Milli-Q A10 ultrapure water device.

XPS (ESCALAB 250, Thermo Fisher Scientific USA), SEM, SEM-EDS (JEM-7800, JEOL, Tokyo, Japan), and NMR (Avance AV 600, Bruker, Mannheim, Germany) were performed in this study.

### 2.2. Verification of Loose Layer on Surface of Zn–Al–Mg Steel Plate

The Zn–Al–Mg steel plate was cleaned three times with petroleum ether, isoamyl acetate, acetone, and ethanol to remove surface oil. The elemental distribution was analyzed with SEM-EDS. The degreased Zn–Al–Mg steel plates were placed in an air-drying oven and dried at 25, 50, 100, 150, 200, 250, and 300 °C for 2 h. Subsequently, XPS was performed at room temperature in a dryer.

The Zn–Al–Mg steel plate was subjected to de-oiling, alkali washing, water washing, and rapid N_2_ atmosphere drying. Subsequently, the cleaned Zn–Al–Mg steel plate was placed in N_2_, O_2_, and H_2_O vapor atmospheres for 48 h, and XPS was immediately performed.

### 2.3. Hydrolysis of Silane Coupling Agents

A silane coupling agent hydrolysate with a Si content of 2.4 × 10^4^ ppm was prepared as follows. A 100 mL three-necked flask was placed in an ice water bath. The flash was added with 20 mL of 0.1 mol/L ammonia, nitric acid, or acetic acid and 20 mL of ethanol and stirred for 10 min; subsequently, 5 mL of KH792 was added to the mixture dropwise. The mixture was stirred for another 3 h, and the reaction equation is as follows. After hydrolysis, the NMR Si (^29^Si-NMR) spectrum of the water-soluble silane coupling agent solution was obtained. 
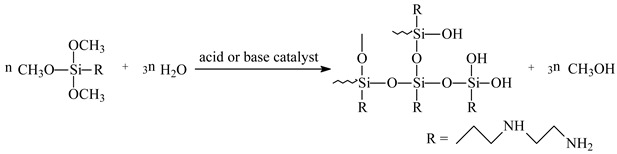


Subsequently, a stability test was performed on the silane coupling agent hydrolysate as follows: the hydrolysate of the silane coupling agent catalyzed by ammonia, nitric acid, acetic acid, or ethanol/water was placed in a cool and dry location for one year before ^29^Si-NMR testing was performed on it.

### 2.4. Adhesive Bonding of Zn–Al–Mg Steel Plate with Epoxy Resin

The epoxy adhesive comprised E51, hexahydrophthalic anhydride, and DMP30 with a mass ratio of 100:93:0.5. The samples were prepared for further utilization by mixing them thoroughly.

The Zn–Al–Mg steel plate (measuring 10 mm × 2 cm × 0.1 cm) was cleaned three times using petroleum ether, isoamyl acetate, acetone, and ethanol to remove surface oil. Subsequently, it was treated with a silane coupling agent hydrolysis solution with Si (300 ppm) and baked in a 200 °C oven for 15 s. Next, it was coated with the prepared epoxy adhesive and placed in a 150 °C oven for 3 h of curing. The cured epoxy resin was peeled off, and SEM-EDS and XPS tests were performed on the sample.

## 3. Results and Discussion

### 3.1. Verification of Loose Layer on Surface of Zn–Al–Mg Steel Plate

After heating and dehydration, hydroxides become oxides, and the absolute oxygen content therein decreases. Based on this concept, if we assume that the loose layer on the surface of the Zn–Al–Mg steel plates is composed of hydroxides, then when the Zn–Al–Mg steel plates are heated, as the temperature increases, hydrogen oxides will become oxides and the amount of O will decrease accordingly, resulting in a loose layer on the surface of the Zn–Al–Mg steel plates. The spatial resolution of XPS equipment is approximately 5 nm, whereas the thickness of the loose layer is approximately 2 nm. Therefore, characterizing the surface element changes in Zn–Al–Mg steel plates using XPS may present significant uncertainties. However, only XPS detection equipment currently has sufficiently high spatial resolutions, and the results obtained via XPS are reasonably satisfactory.

**Figure 1:**
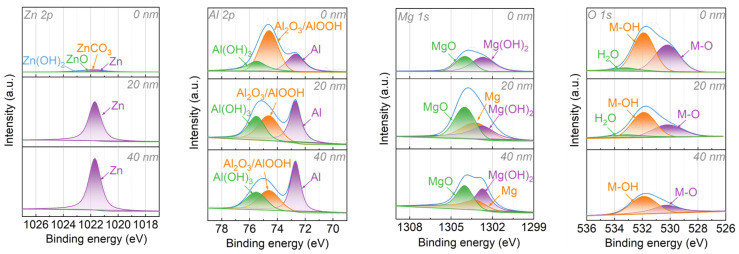
Valence forms of surface depth elements in different Zn–Al–Mg steel plates.

XPS utilizes monochromatic Al Ka radiation (hv = 1486.6 eV, 225 W) as the X-ray source, and all spectra are calibrated by C 1s (284.8 eV). Utilizing XPS analysis equipment and argon ion etching, the valence and content changes of surface elements in the Zn–Al–Mg steel plates at different depths were studied. Figure 1 illustrates the XPS analysis results of the original surface of the Zn–Al–Mg steel plate after etching at 20 and 40 nm. According to Figure 1, as the etching depth increases, the oxygen content decreases, and Al and Mg gradually change from hydroxide to oxide and metal. Simultaneously, Zn changes from hydroxide and oxide to metal. The steel plate underwent pit leveling during the manufacturing process, and several micrometer-deep pits are present on its surface, which has a certain impact on the XPS etching fault analysis. However, the overall analysis results are reasonable, indicating a large amount of hydroxide on the surface of the Zn–Al–Mg steel plate.

**Figure 2:**
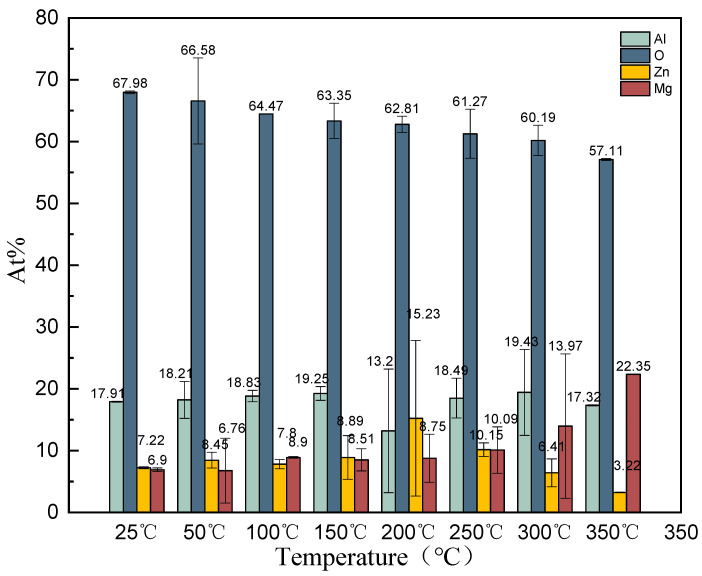
Atomic content ratio of surface elements on Zn–Al–Mg steel plate after heat treatment at different temperatures.

Changes in the Zn, Al, Mg, and O contents on the surface of the Zn–Al–Mg steel plates after different temperature treatments were investigated using XPS, and the results are illustrated in Figure 2. The vertical axis in Figure 2 represents the proportion of atoms in each element (at.%), whereas the horizontal axis represents the different processing temperatures. The XPS analysis results demonstrate that the atomic content of O on the surface of the Zn–Al–Mg steel plate was extremely high. As the heat temperature increased, the atomic O content decreased gradually. Before and after heat treatment, the atomic content of Zn did not change significantly, whereas the atomic proportions of Al and Mg increased with the treatment temperature. The loose layer was composed of magnesium hydroxide and aluminum hydroxide. As the heat treatment temperature increased, the hydroxide dehydrated and transformed into an oxide. O was lost in the form of water molecules, and the atomic proportions of Al and Mg increased. The atomic proportion of Zn in the Zn–Al–Mg steel plate did not change, thus indicating a relatively low content of zinc hydroxide in the loose layer. Therefore, the atomic proportion of Zn did not change significantly before and after the heat treatment. This indirectly indicates that the Mg and Al in the Zn–Al–Mg steel plates were first oxidized as sacrificial elements and formed a loose layer to protect the steel plate from further oxidation.

A loose layer formed by magnesium hydroxide and aluminum hydroxide was present on the surface of the Zn–Al–Mg steel plate, effectively preventing further oxidation and corrosion by water and oxygen in the air and thus protecting the Zn–Al–Mg steel plate from oxidation. Consequently, the anti-corrosion effect of the coated Zn–Al–Mg steel plate was much higher than that of the galvanized steel plate. However, because of the weak intermolecular force in the loose layer and the weak bonding force with the steel plate, when a Zn–Al–Mg steel plate is utilized as an automobile plate for bonding, the adhesive only reacts with the surface of the loose layer, whereas the internal loose structure and the low force between the loose layer and steel plate prevent the transfer of external force. When impacted by external forces, the bonding interface is typically damaged, thus resulting in debonding. Therefore, the anti-corrosion performance of Zn–Al–Mg steel plates is much better than that of galvanized steel plates, and the bonding performance of automobile plates is inferior to that of galvanized steel plates. This is attributable to the loose layer of sheet-like structures composed of magnesium hydroxide and aluminum hydroxide on the surface of the Zn–Al–Mg steel plate.

### 3.2. Causes of Loose Layer on Surface of Zn–Al–Mg Steel Plate

The excellent corrosion resistance of the Zn–Al–Mg steel plate is attributed to the loose layer on its surface. The surface of commercial Zn–Al–Mg steel plates has a protective oil film that can be dried rapidly under N_2_ protection through oil removal, alkaline solution washing, water washing, and N_2_ protection, which remove the oil film and loose surface layer on Zn–Al–Mg steel plates. Clean Zn–Al–Mg steel plates were placed in N_2_, O_2_, and saturated H_2_O atmospheres for 48 h and then subjected to a rapid XPS analysis to investigate the atomic content changes of Zn, Al, Mg, and O. The high proportion of the atomic content of O indicates a high content of hydroxide and a thicker loose layer, suggesting that intermediate conditions are more likely to cause oxidation on the surface of the Zn–Al–Mg steel plates, thus rendering it easier to form a loose layer.

**Figure 3:**
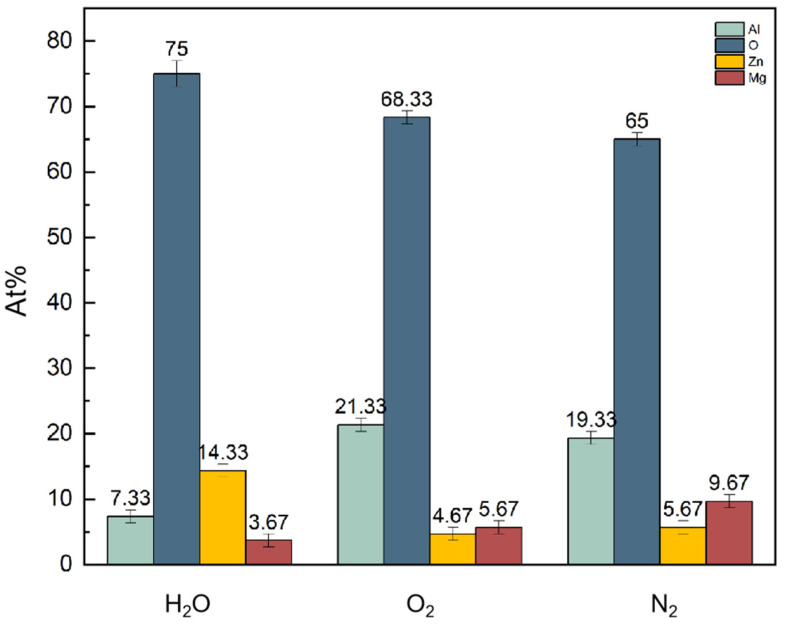
Surface element at.% ratio of clean Zn–Al–Mg steel plate in N_2_, O_2_, and H_2_O atmospheres.

Figure 3 illustrates the atomic content ratios of the main elements on the surface of clean Zn–Al–Mg steel plates, as determined via XPS under N_2_, O_2_, and H_2_O atmospheres. Theoretically, under an N_2_ atmosphere, oxidation does not occur on the surface of the Zn–Al–Mg steel plates; however, the O content was extremely high, thus indicating that oxidation had already happened during the preparation of clean Zn–Al–Mg steel plates. Water cleaning was performed to prepare clean Zn–Al–Mg steel plates. Although the operation time was short, some oxidation reactions were observed in the XPS results. Figure 3 demonstrates that after 48 h in N_2_, O_2_, and H_2_O atmospheres, the elemental content on the surface of the Zn–Al–Mg steel plates changed significantly. The atomic content of O in the H_2_O and O_2_ atmospheres was substantially higher than that in the N_2_ atmosphere, indicating that O_2_ and H_2_O vapors significantly affected the formation of loose layers. Moreover, the loose layer formed on the surface of the Zn–Al–Mg steel plates via water vapor was thicker than that formed in an O_2_ atmosphere. The atomic proportion of O_2_ on the surface of clean Zn–Al–Mg steel plates under a H_2_O vapor atmosphere was the highest, i.e., 75%.

**Figure 4:**
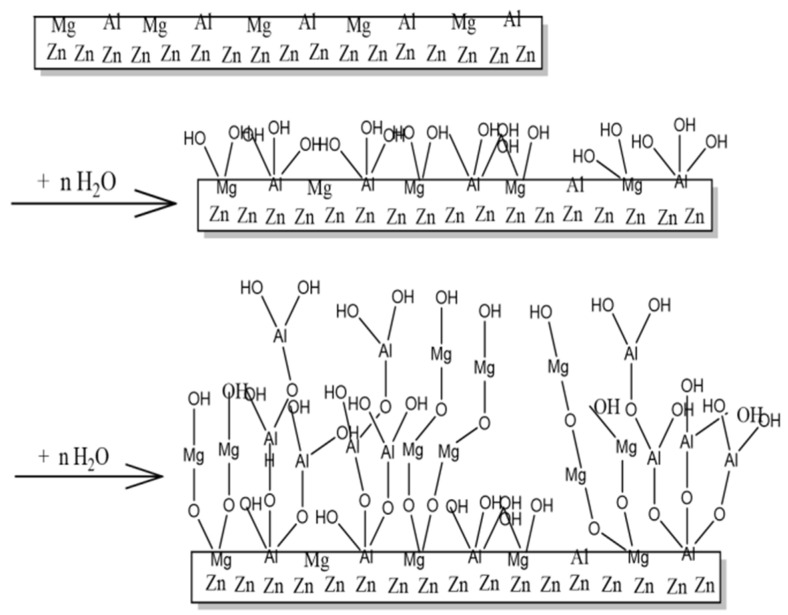
Schematic illustrating formation of loose layer on surface of Zn–Al–Mg steel plate in air.

The surface O content of the Zn–Al–Mg steel plate under the O_2_ atmosphere was higher than that under the N_2_ atmosphere; however, it was not comparable to the increase in O content under the H_2_O atmosphere, indicating that the formation of loose layers was mainly due to H_2_O. A schematic of the reaction between active Al and Mg on the surface of the Zn–Al–Mg steel plate and H_2_O in the air to generate hydroxides is illustrated in Figure 4. The metal and H_2_O in the air underwent a single displacement reaction [M + 2H_2_O = M(OH)_2_ + H_2_] to generate magnesium hydroxide and aluminum hydroxide sacrificial layers. A single displacement reaction of H_2_O occurred on the Zn–Al–Mg steel plate’s surface, forming a loose layer of flake hydroxide. Although this hydroxide layer is loose, a loose layer with a thickness of several nanometers is sufficient to prevent further single-displacement reactions and corrosion on the steel plate.

The reaction between O_2_ and Al or Mg on the surface of the Zn–Al–Mg steel plate is a gas–solid reaction, and the reaction efficiency is extremely low from the perspective of reaction kinetics. Although the content of O_2_ in the air is high, only a trace amount of Al or Mg is directly oxidized with O_2_ to generate stable oxides. The reaction of H_2_O with Mg or Al on the surface of a Zn–Al–Mg steel plate is a liquid–solid reaction. H_2_O is adsorbed on the surface to produce hydroxide and hydrogen through a single displacement reaction. Although the content of H_2_O in air is low, provided that H_2_O is present, it will condense on the surface of the steel plate, and a single displacement reaction will occur with the Al or Mg on its surface. These hydroxides form a scaly morphology on the surface of the Zn–Al–Mg steel plates. They prevent further reactions between water and Al or Mg when they reach a certain thickness. Therefore, Al and Mg exhibit self-protective functions in the air.

### 3.3. Element Distribution on Surface of Zn–Al–Mg Steel Plate

**Figure 5:**
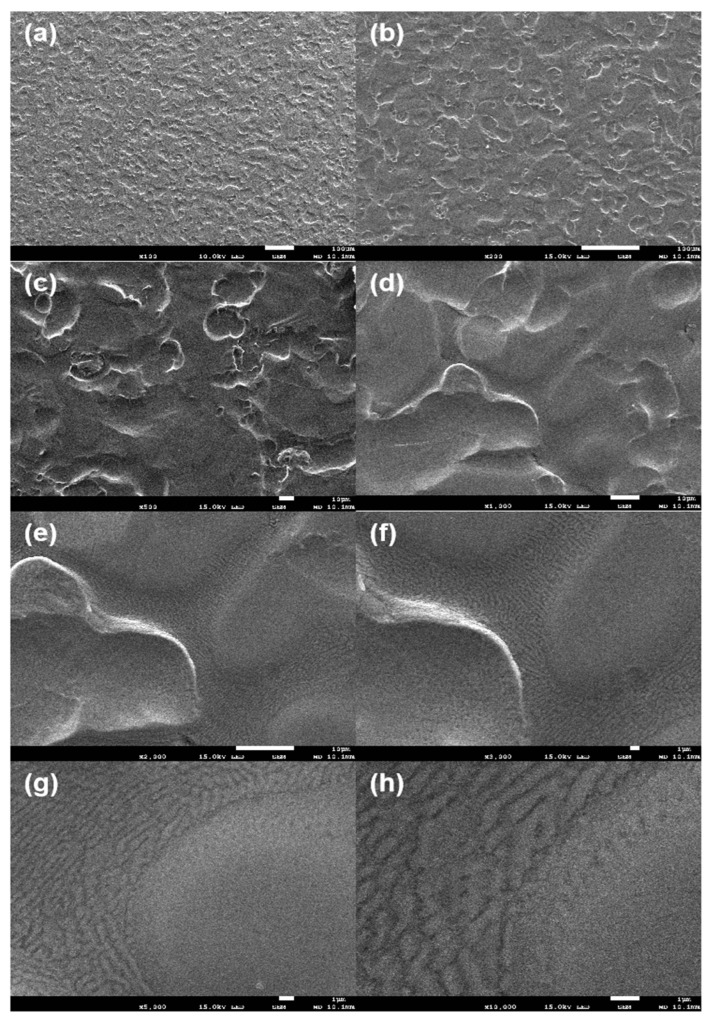
SEM images of surface of Zn–Al–Mg steel plate ((**a**–**h**) represent different magnifications).

Figure 5 illustrates the SEM image of a degreased Zn–Al–Mg steel plate. The SEM images illustrate that pit treatment was performed during the steel plate preparation process to improve bonding performance. In addition, the surface grain boundaries and the grain boundaries between the Zn single crystals demonstrated many regular scale-like morphologies speculated to be hydrogen oxides.

**Figure 6:**
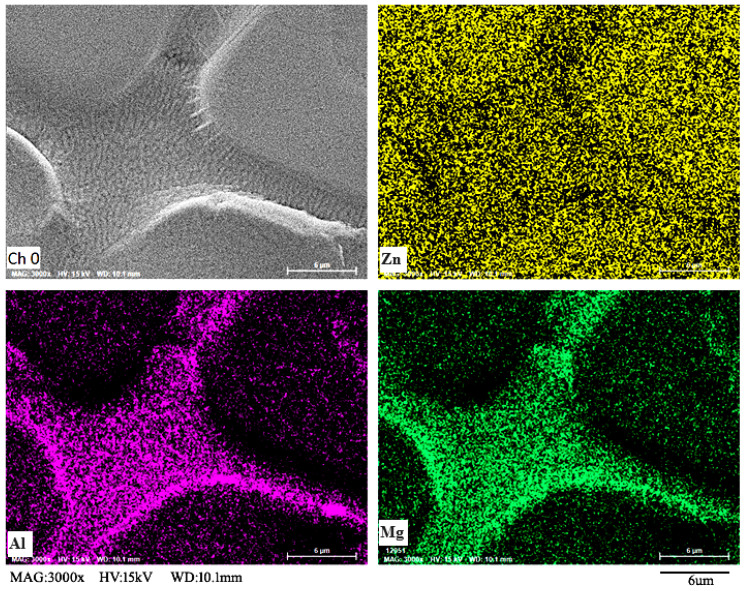
Element surface distribution on surface of Zn–Al–Mg steel plate obtained via SEM-EDS.

Figure 6 illustrates SEM-EDS diagrams of Zn, Al, and Mg on the surface of the degreased Zn–Al–Mg steel plate. Based on the elemental surface distribution diagram, the distribution of Zn was relatively uniform, and the contents of Al and Mg were relatively low and uneven on the material surface. Enrichment was indicated at the grain boundary, indicating that Mg and Mg migrated during the cooling crystallization of the coating. The oxidation potentials of Al and Mg are extremely low, allowing them to react easily with water in the air. Therefore, Al and Mg can react with water in the air at the grain boundaries to form magnesium hydroxide and aluminum hydroxide scales.

### 3.4. Hydrolysis of Silane Coupling Agents

**Figure 7:**
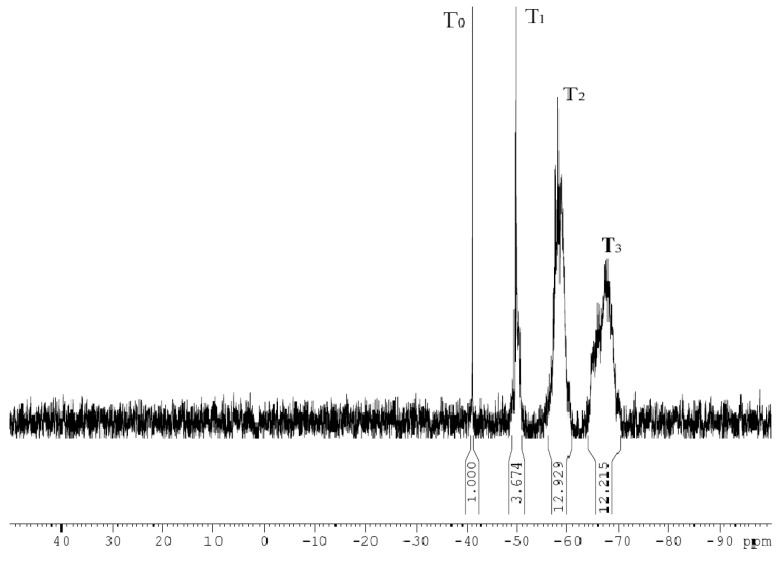
^29^Si-NMR spectrum of silane coupling agent hydrolysate.

Figure 7 illustrates the ^29^Si-NMR spectrum of the silane coupling agent hydrolysate with a Si content of 2.4 × 10^4^ ppm (KH792 hydrolyzed in a 50% ethanol system), which was employed to analyze the structural changes after the hydrolysis of the silane coupling agent based on the ^29^Si chemical shift and peak area. The chemical shift assignment of the ^29^Si spectrum revealed that T_0_ was connected only to the silicon hydroxyl groups on the Si atom, T_1_ was connected to one Si-O-Si on the Si atom, T_2_ was connected to two Si-O-Si atoms on the Si atom, and T_3_ was connected to three Si-O-Si groups on the Si atom. The chemical shifts corresponding to T_0_, T_1_, T_2_, and T_3_ were approximately 41, 49, 58, and 67 ppm, respectively, whereas the peak integral area ratio of ^29^Si to T_0_, T_1_, T_2_, and T_3_ was 1:4:13:12.

**Figure 8:**
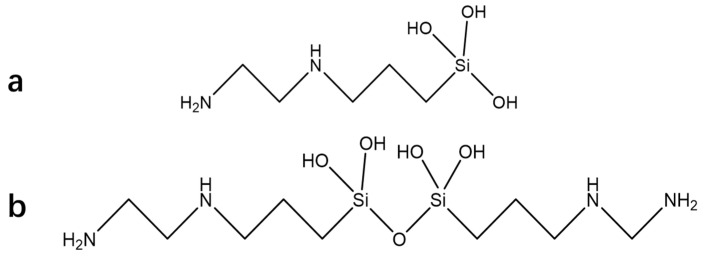
Single-molecule configuration (**a**) and dimer configuration (**b**) of KH792 hydrolysis.

The hydrolysis of silane coupling agents generates different aggregation states. The molecular weight distribution of the hydrolysis structures of the silane coupling agents was determined from the chemical shift and peak integral area of Si in the ^29^Si-NMR spectrum. The hydrolysis of silane coupling agent KH792 generated aggregation states with different structures, and structures T_0_, T_1_, T_2_, and T_3_ were numbered based on the number of Si atoms connecting the OH and Si-O-Si bridge bonds. Figure 8 illustrates the monomolecular and dimeric structures generated by the hydrolysis of KH792. In the monomolecular state, only a T_0_ (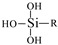) chemical shift was observed in the Si atom, whereas in the dimeric state, a T_1_ (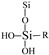) chemical shift was observed in the Si atom.

**Figure 9:**
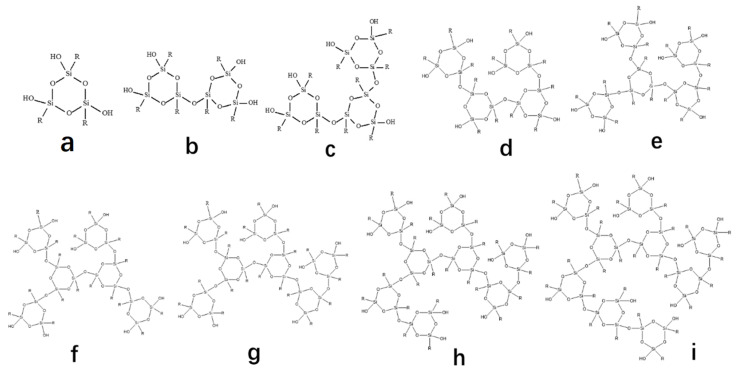
Cyclic polymer configuration of KH792 hydrolysis ((**a**–**i**) correspond to one to nine cycles, respectively; R = CH_2_CH_2_CH_2_NHCH_2_CH_2_NH_2_).

The different aggregation states that occurred after the hydrolysis of KH792 resulted in the slowest hydrolysis of the first alkoxy chain of the silane coupling agent during the hydrolysis process. After the first hydroxyl group was generated, the other alkoxy groups accelerated hydrolysis, making it difficult to control the hydrolysis rate. During the hydrolysis of KH792, different molecular configurations were formed through cyclization polymerization. Three silane coupling agents were cyclized to form a six-membered ring structure, and several six-membered ring structures were polymerized to form macromolecular aggregates. The aggregate structures contained different amounts of T_2_ (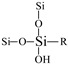) and T_3_ (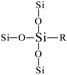). After hydrolysis, the silane coupling agent formed a polymer structure. A silane coupling agent with a 1–9 ring structure is illustrated in Figure 9 (where R = CH_2_CH_2_CH_2_NHCH_2_CH_2_NH_2_). In the different aggregates, the T_2_ and T_3_ types of Si constituted different proportions of the integrated area. Figure 9a illustrates that Si exhibited only the T_2_ structure. Meanwhile, Figure 9b illustrates T_2_:T_3_ = 4:2; Figure 9c, T_2_:T_3_ = 5:4; Figure 9d, T_2_:T_3_ = 6:6; Figure 9e, T_2_:T_3_ = 7:8; Figure 9f, T_2_:T_3_ = 8:10; Figure 9g, T_2_:T_3_ = 9:12; Figure 9h, T_2_:T_3_ = 10:14; and Figure 9i, T_2_:T_3_ = 11:16. These hydrolysis structural ratios demonstrate that the higher the proportion of the T_3_ integral area in the ^29^Si spectrum, the larger the aggregate hydrolyzed by the silane coupling agent. Therefore, the molecular weight of the silane coupling agent after hydrolysis was determined by using ^29^Si-NMR spectroscopy.

**Figure 10:**
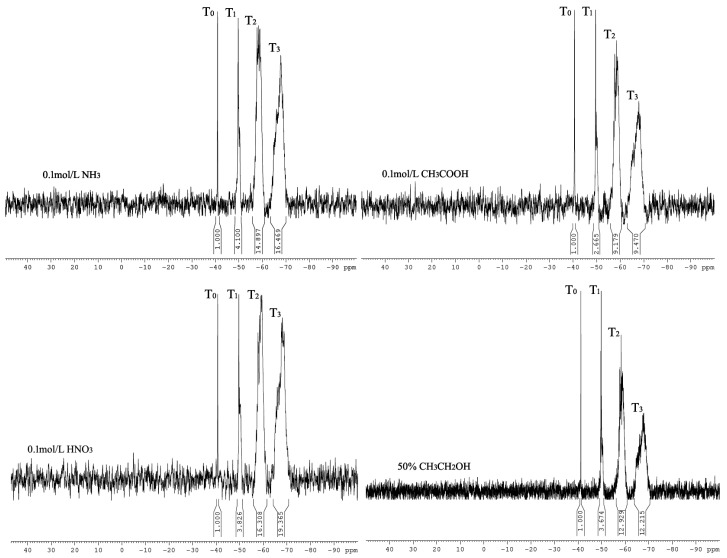
^29^Si-NMR spectra of silane coupling agent hydrolysate with Si element content of 2.4 × 10^4^ ppm under different hydrolysis medium conditions.

The molecular weight of a silane coupling agent after hydrolysis determines its applicability. The molecular structure of the silane coupling agent can be controlled by controlling the hydrolysis conditions. Here, nitric acid, acetic acid, ammonia, and ethanol/water were selected as the hydrolysis solvents for KH792, and the effect of the catalyst system in the hydrolysis medium on the molecular weight of the silane coupling agent was investigated. Figure 10 illustrates the ^29^Si-NMR spectra of the silane coupling agent hydrolysate with a Si content of 2.4 × 10^4^ ppm. The hydrolysis media of the silane coupling agent were 0.1 mol/L ammonia, 0.1 mol/L acetic acid, 0.1 mol/L nitric acid, and 50% ethanol. Figure 10 illustrates that the T_3_ integral area of the ^29^Si spectrum was slightly larger when the silane coupling agent was hydrolyzed under strongly acidic medium conditions, indicating that a strong catalyst tends to generate larger silane coupling agent hydrolysis aggregates. Whether in an ethanol medium or a weakly acidic or weakly alkaline medium, the T_2_ and T_3_ integral areas of the ^29^Si spectrum were equivalent, and the main hydrolysis structure is illustrated in Figure 9d,e as four and five polycyclic structures, respectively. The hydrolytic structures of the silane coupling agents were relatively small.

**Figure 11:**
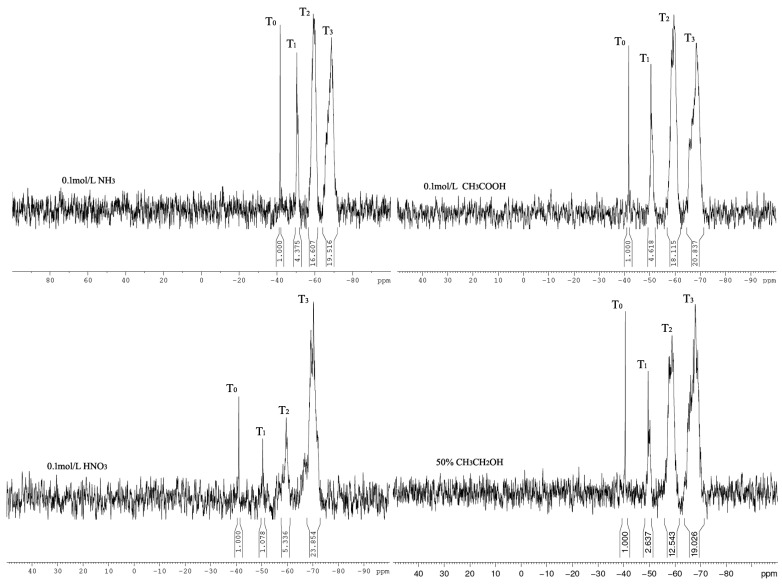
^29^Si-NMR spectra of silane coupling agent hydrolysate with Si element content of 2.4 × 10^4^ ppm under different hydrolysis medium conditions after one year of storage.

The storage stability of silane coupling agent hydrolysate affects the repeatability of its usage. The silane coupling agent hydrolysates of the ammonia, acetic acid, nitric acid, and ethanol/water systems were placed in a cool and dry location for one year and then subjected to ^29^Si-NMR testing (Figure 11). The integrated area of T_3_ in the ^29^Si spectrum increased; however, no significant change was indicated in terms of the integrated area or peak shape of the T_1_, T_2_, and T_3_ positions of the silane coupling agent hydrolysate in the ammonia–water, acetic acid, and ethanol/water systems, indicating that the hydrolysis structure of the silane coupling agent remained unchanged. The integral area of the T_3_-type Si hydrolysis solution of the silane coupling agent in a dilute nitric acid medium increased significantly, indicating that the silane coupling agent underwent aggregation during placement to form larger aggregate structures. Although no apparent precipitation was indicated, the coupling agent could not easily penetrate and reinforce the interior of the loose layer. Therefore, the hydrolysis solution of the silane coupling agent can be stably placed in weakly alkaline, weakly acidic, and neutral media, whereas strongly acidic media cannot be stably placed for a long time.

### 3.5. Modification of Zn–Al–Mg Steel Plate

**Figure 12:**
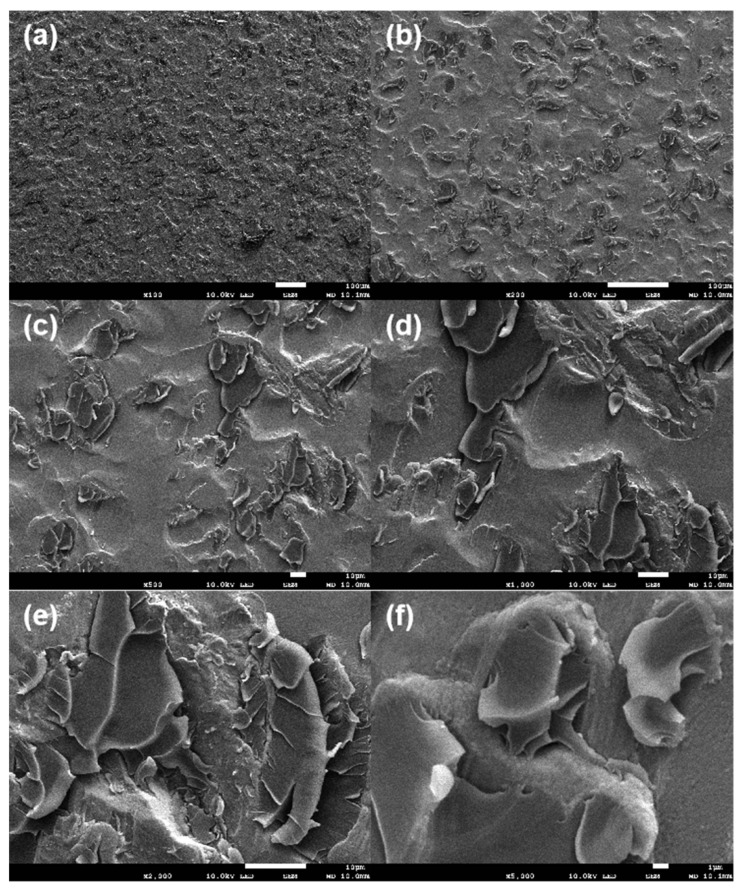
SEM image of surface of modified Zn–Al–Mg steel plate after epoxy resin bonding ((**a**–**f**) represent different magnifications).

The silane coupling agent was hydrolyzed and prepared as a treatment solution with a Si content of 300 ppm. The Zn–Al–Mg steel plate was modified using a specific coating method. After the silane coupling agent-modified Zn–Al–Mg steel plate underwent a bonding reaction with the epoxy resin, the epoxy resin was peeled off from the steel plate interface under liquid nitrogen freezing conditions. The surface morphology and elemental distribution of the Zn–Al–Mg steel plate were examined utilizing SEM and SEM-EDS. The SEM image illustrated the presence of residual epoxy resin in the pits of the steel plate and at the grain boundaries of Zn, indicating that the silane modifier effectively strengthened the loose layer on the Zn–Al–Mg plate (see Figure 12). Tensile and shear tests were performed on the epoxy resin bonding after the surface of the Zn–Al–Mg steel plate was modified. After the surface modification, the fracture strength of the steel plate increased, and the main fracture was the structural cracking of the epoxy resin.

**Figure 13:**
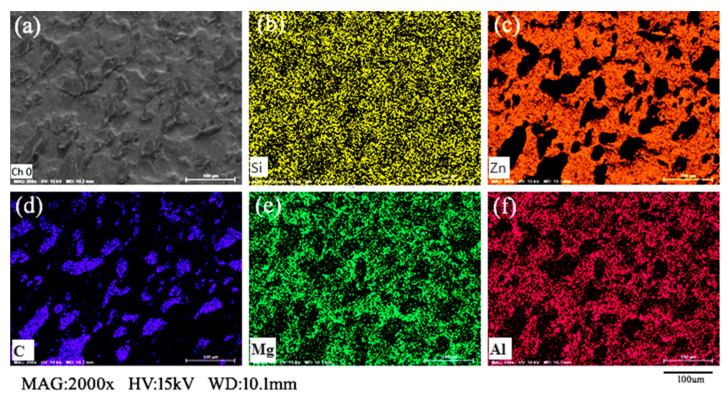
SEM-EDS result illustrating element distribution on surface of modified Zn–Al–Mg steel plate after epoxy resin bonding ((**a**) is the SEM diagram; (**b**–**f**) illustrate the element distributions of Si, Zn, C, Mg, and Al, respectively).

Figure 13 illustrates an SEM-EDS image of the surface of the Zn–Al–Mg steel plate after the epoxy resin adhesive was peeled off. As illustrated, the distributions of Zn, Al, and Mg on the surface of the modified Zn–Al–Mg steel plate remained unchanged, whereas the distribution of Si was uniform, thus indicating that the silane coupling agent was uniformly coated on the surface of the steel plate. However, the distribution of C was uniform throughout the body and locally clustered. At the pits and grain boundaries on the surface of the Zn–Al–Mg steel plates, the bonding strength with the epoxy resin was higher owing to the modification of the silane coupling agents. When the epoxy resin structure fractured, some epoxy resin remained on the surface of the Zn–Al–Mg steel plates.

### 3.6. Adhesion Performance of Zn–Al–Mg Steel Plate

To verify the effectiveness of the silane coupling agent in the surface modification of the Zn–Al–Mg steel plates, the silane coupling agent was employed to reinforce the loose layer on their surface. The bonding between the Zn–Al–Mg steel plate and a typical commercial automobile adhesive was evaluated by adopting the national standard method (GB/T 7124-2008, Determination of Tensile Shear Strength of Adhesives).

In Table 1, A, B, C, D, E, and F represent six typical commercial automotive spot welding and shock absorption sealant products whose adhesion performance is inferior to that of the Zn–Al–Mg steel plate. Table 1 compares the results of bonding experiments of Zn–Al–Mg steel plates with several commercial automotive adhesives before and after modification. As demonstrated, several typical automotive adhesives caused significant changes to the bonding tensile strength of the Zn–Al–Mg steel plates before and after modification. Before the Zn–Al–Mg steel plates were modified with silane coupling agents, interfacial cracking occurred in the steel plates owing to tensile shear fracture, whereas after modification, tensile shear fracture occurred owing to the structural damage of the adhesive layer. This proves that the silane coupling agents reinforced the loose surface layer of the Zn–Al–Mg steel plates.

**Figure 14:**
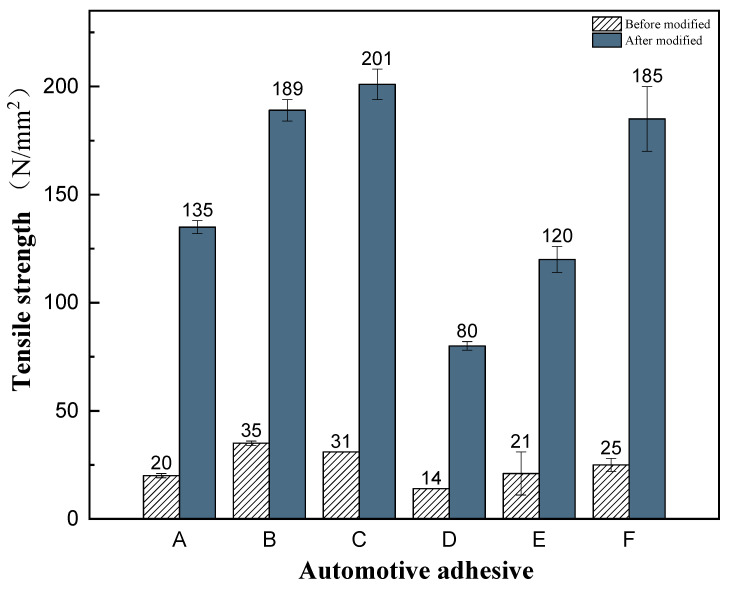
Tensile and shear strength of Zn–Al–Mg steel plate and vehicle adhesive before and after modification (A, B, C, D, E, and F represent different brands of commercial automotive adhesives).

Figure 14 illustrates the tensile and shear test results of the six automotive adhesives before and after the modification of the Zn–Al–Mg steel plates. Based on the results, the surface modification of the Zn–Al–Mg steel plates significantly enhanced the bonding strength, indicating that the silane coupling agent effectively strengthened the loose surface layer on the Zn–Al–Mg steel plates, thereby enabling the bonding interface to transfer stress effectively and consequently improving the tensile strength significantly.

## 4. Conclusions

The change in O content on the surface of the Zn–Al–Mg steel plate with respect to the treatment temperature was investigated via XPS. As the treatment temperature increased, the hydroxide dehydrated to transform the oxide, which manifested as a decrease in the original oxygen content in the XPS result. Therefore, we concluded that a weak, loose layer composed of hydroxide was formed on the surface of the Zn–Al–Mg steel plate. The change in oxygen content on the surface of the Zn–Al–Mg steel plate with the oxide film removed after being placed in H_2_O vapor, O_2_, and N_2_ atmospheres for 48 h confirmed that the loose layer was mainly composed of Al, Mg, and water, which underwent a single displacement reaction to generate Al(OH)_3_ and Mg(OH)_2_. SEM and SEM-EDS analyses revealed that Mg and Al on the surface of the Zn–Al–Mg steel plates were enriched near the grain boundaries of the Zn single crystals and formed a scale-like structure.

The molecular weights and structural morphologies of the hydrolysis products of the silane coupling agents were investigated via ^29^Si-NMR spectroscopy. The T_0_, T_1_, T_2_, and T_3_ structural forms of Si in the ^29^Si-NMR spectrum and the molecular structure of the silane coupling agent products that may have been formed from these structural forms were quantitatively investigated. In a strongly acidic medium, the molecular structure of the silane coupling agent hydrolysate was larger, whereas smaller molecular structures were easier to obtain via the catalytic hydrolysis of weak acids or bases. Meanwhile, under strongly acidic medium conditions, the storage stability of the silane coupling agent hydrolysates was unsatisfactory, whereas in weakly alkaline, neutral, and weakly acidic media, the silane coupling agent hydrolysates exhibited high storage stability. After diluting the hydrolysate of the silane coupling agent to a Si content of 300 ppm, a modified Zn–Al–Mg steel plate was coated. The loose layer on the Zn–Al–Mg steel plate’s surface was reinforced, resulting in a strong and dense film. The bonding strength of typical automotive adhesives has improved significantly, and the results of tensile and shear tests revealed that the failure of the bonding joint was 90–100% of the cohesive failure of the adhesive. This provides an effective solution for improving the surface bonding performance of Zn–Al–Mg steel plates via higher corrosion resistance, allowing the application of Zn–Al–Mg steel plates to be further expanded. Furthermore, the results of this study provide novel ideas for material modification schemes.

## Figures and Tables

**Table 1:** Comparison of bonding effects between Zn–Al–Mg steel plate and automotive adhesive before and after modification.

Type of Adhesive	Modification or Not	Morphology of Tensile Shear Fracture Interface	Fracture Ratio of Adhesive Interface
A	×	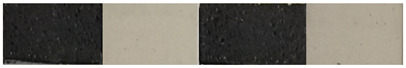	0%
	√	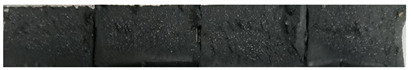	95%
B	×	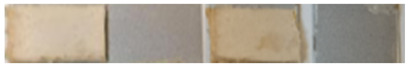	0%
	√	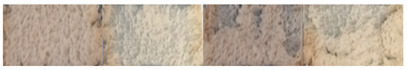	100%
C	×	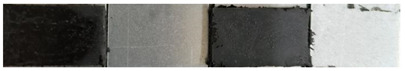	0%
	√	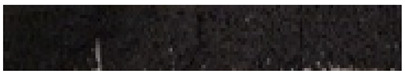	100%
D	×	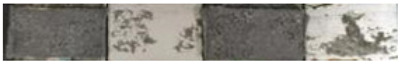	10%
	√	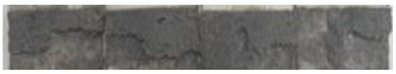	100%
E	×	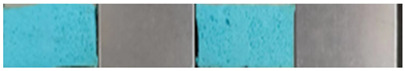	0%
	√	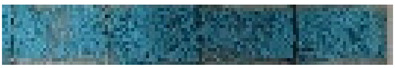	95%
F	×	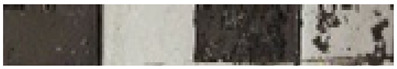	0%
	√	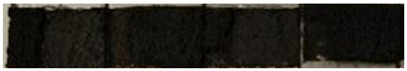	100%

## Data Availability

All data included in this study are available upon request by contact with the corresponding author.
